# The Kilim plot: A tool for visualizing network meta‐analysis results for multiple outcomes

**DOI:** 10.1002/jrsm.1428

**Published:** 2020-07-16

**Authors:** Michael Seo, Toshi A. Furukawa, Areti Angeliki Veroniki, Toby Pillinger, Anneka Tomlinson, Georgia Salanti, Andrea Cipriani, Orestis Efthimiou

**Affiliations:** ^1^ Institute of Social and Preventive Medicine University of Bern Bern Switzerland; ^2^ Departments of Health Promotion and Human Behavior and of Clinical Epidemiology Kyoto University Graduate School of Medicine/School of Public Health Kyoto Japan; ^3^ Department of Primary Education, School of Education University of Ioannina Greece; ^4^ Knowledge Translation Program Li Ka Shing Knowledge Institute, St. Michael's Hospital, Unity Health Toronto Toronto Ontario Canada; ^5^ Institute of Reproductive and Developmental Biology, Department of Surgery & Cancer, Faculty of Medicine Imperial College London London UK; ^6^ Institute of Psychiatry, Psychology and Neuroscience King's College London London UK; ^7^ MRC London Institute of Medical Sciences, Faculty of Medicine Imperial College London London UK; ^8^ Department of Psychiatry University of Oxford Oxford UK; ^9^ Oxford Health NHS Foundation Trust Warneford Hospital Oxford UK

**Keywords:** indirect comparisons, mixed evidence, multiple outcomes, multiple treatments meta‐analysis, visualization

## Abstract

Network meta‐analysis (NMA) can be used to compare multiple competing treatments for the same disease. In practice, usually a range of outcomes is of interest. As the number of outcomes increases, summarizing results from multiple NMAs becomes a nontrivial task, especially for larger networks. Moreover, NMAs provide results in terms of relative effect measures that can be difficult to interpret and apply in every‐day clinical practice, such as the odds ratios. In this article, we aim to facilitate the clinical decision‐making process by proposing a new graphical tool, the Kilim plot, for presenting results from NMA on multiple outcomes. Our plot compactly summarizes results on all treatments and all outcomes; it provides information regarding the strength of the statistical evidence of treatment effects, while it illustrates absolute, rather than relative, effects of interventions. Moreover, it can be easily modified to include considerations regarding clinically important effects. To showcase our method, we use data from a network of studies in antidepressants. All analyses are performed in R and we provide the source code needed to produce the Kilim plot, as well as an interactive web application.

**What is already known?**

- Network meta‐analysis (NMA) can be used to compare multiple competing treatments for the same disease. A range of outcomes is usually of interest in a NMA.
- It can be difficult to efficiently summarize results from NMAs on many outcomes, especially when the number of treatments in the network and the number of outcomes is large.
- NMAs often provide results in terms of relative effect measures that can be difficult to apply in every‐day clinical practice, such as the odds ratios.

**What is new?**

- We propose a new graphical tool, the “Kilim plot,” aiming to facilitate the clinical decision‐making process.
- The Kilim plot can be used to visualize results from NMA on multiple outcomes. It provides information regarding the strength of statistical evidence of treatment effects, and it illustrates absolute, rather than relative, effects of interventions.
- The plot can include considerations regarding clinically important effects.

**Potential impact for RSM readers outside the authors' field**

- The Kilim plot can be a valuable aid in visualizing results from NMAs on multiple outcomes.
- It can be especially useful for larger networks, for the case of many outcomes, and when aiming to communicate NMA results with patients and/or clinicians, so as to facilitate every‐day clinical practice.

## 1 INTRODUCTION

Network meta‐analysis (NMA) can be used to synthesize direct and indirect evidence among many competing interventions so as to guide clinical decisions.^1^ In practical applications of NMA there are usually many outcomes of interest which play a role in the decision‐making process.^2^ However, effectively summarizing results from NMAs on multiple outcomes can be challenging. The usual methods of presenting NMA results, that is, showing relative effects vs a reference treatment or via the so‐called “league tables,” become difficult to use as the number of treatments and outcomes increase.^3^ In such cases, implementing NMA results in clinical practice can be very difficult.

Veroniki et al^4^ developed a heat plot aiming to facilitate the visualization of results obtained from multiple outcomes NMA. To this aim, they used the results of ranking statistics for interventions included in a NMA, such as the surface under the cumulative ranking curve (SUCRA).^5^ After fitting a NMA, a ranking statistic is calculated for each outcome and then, using a color scheme, results on all outcomes and treatments are compactly visualized. The plot facilitates the identification of treatments that perform well across all outcomes. Pillinger et al^6^ presented a similar plot in a NMA for antipsychotics on metabolic function in patients with schizophrenia. A drawback of this approach is that ranking statistics (ie, SUCRAs) do not have a straightforward clinical interpretation, in contrast to estimated effect sizes and their corresponding strength of statistical evidence; the latter are arguably clinically more useful. Veroniki et al^7^ presented a rank‐heat plot using the number needed to treat (NNT); however, the use of NNT has been sometimes criticized.^8^ Law et al^9^ used effect sizes and *P*‐values to visualize the network diagram; their approach, however, may be impractical to use for the case of many outcomes.

Moreover, NMA results are usually presented in terms of relative effects.^10^ This, however, can be problematic, especially for binary outcomes. This is because clinical significance hinges more on absolute outcomes, that is, regarding the risk of an event occurring; or, in terms of risk differences, rather than on odds ratios or risk ratios. The latter are commonly used in fitting NMA, due to their superior statistical properties, but may be hard to use for practical decision‐making purposes. In addition, clinicians may also be interested in incorporating clinically important values in their decisions. This is the case, for example, when due to additional costs it is deemed worthwhile to prescribe a new intervention when it increases the probability of response by 10% or more as compared to treatment as usual; or when the probability of a side effect is increased by no more than 5% as compared to the corresponding probability in placebo. In such cases, published results from NMAs may not be very informative, so as to guide decision among competing drugs.

We hereby set out to develop a novel graphical tool, the “Kilim plot” (named after a type of colorful rugs). Our predefined goals when designing this tool were the following: (a) to provide meta‐analysts with a method for summarizing and visualizing the evidence from multiple outcomes NMA; (b) to present results in terms of absolute rather than relative effects; (c) to illustrate graphically the evidence with respect to clinically important values.

To illustrate our tool we use a dataset that compares antidepressants with respect to nine outcomes related to treatment tolerability. We provide all R codes needed to produce the graph in the Appendix and online (https://github.com/MikeJSeo/phd/tree/master/kilim). Finally, we illustrate the Kilim plot via an interactive web application (https://cinema.ispm.unibe.ch/shinies/kilim/).

## 2 ILLUSTRATIVE EXAMPLE: TOLERABILITY OF SEVEN ANTIDEPRESSANTS ACCORDING TO NINE OUTCOMES

The dataset was based on a sample of 297 randomized controlled trials about antidepressants for the acute treatment of depression,^11^ using a predefined protocol.^10^ In this working example, the studies compared seven antidepressants and placebo with respect to nine different side effects (all binary outcomes). Three of the outcomes were serious side effects, that is, suicidal ideation, aggression, and accidental overdose. The other six outcomes corresponded to more frequent side effects, that is, nausea, headache, dry mouth, insomnia, sexual dysfunction, and diarrhea. According to the original protocol,^10^ the ultimate aim is to compare 21 drugs across more than 35 outcomes. We hereby only use a subset of the entire dataset, only for methodological purposes. To avoid a potential use of our example in clinical practice, and also due to confidentiality issues with respect to data, all drug names have been anonymized in all results we show in this article.

**FIGURE 1:**
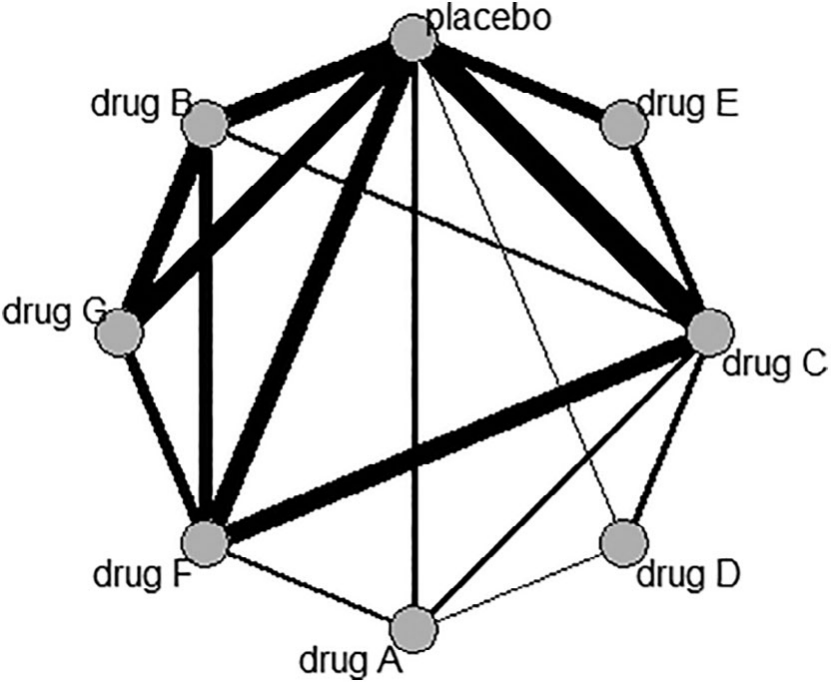
Network graph for nausea

**FIGURE 2:**
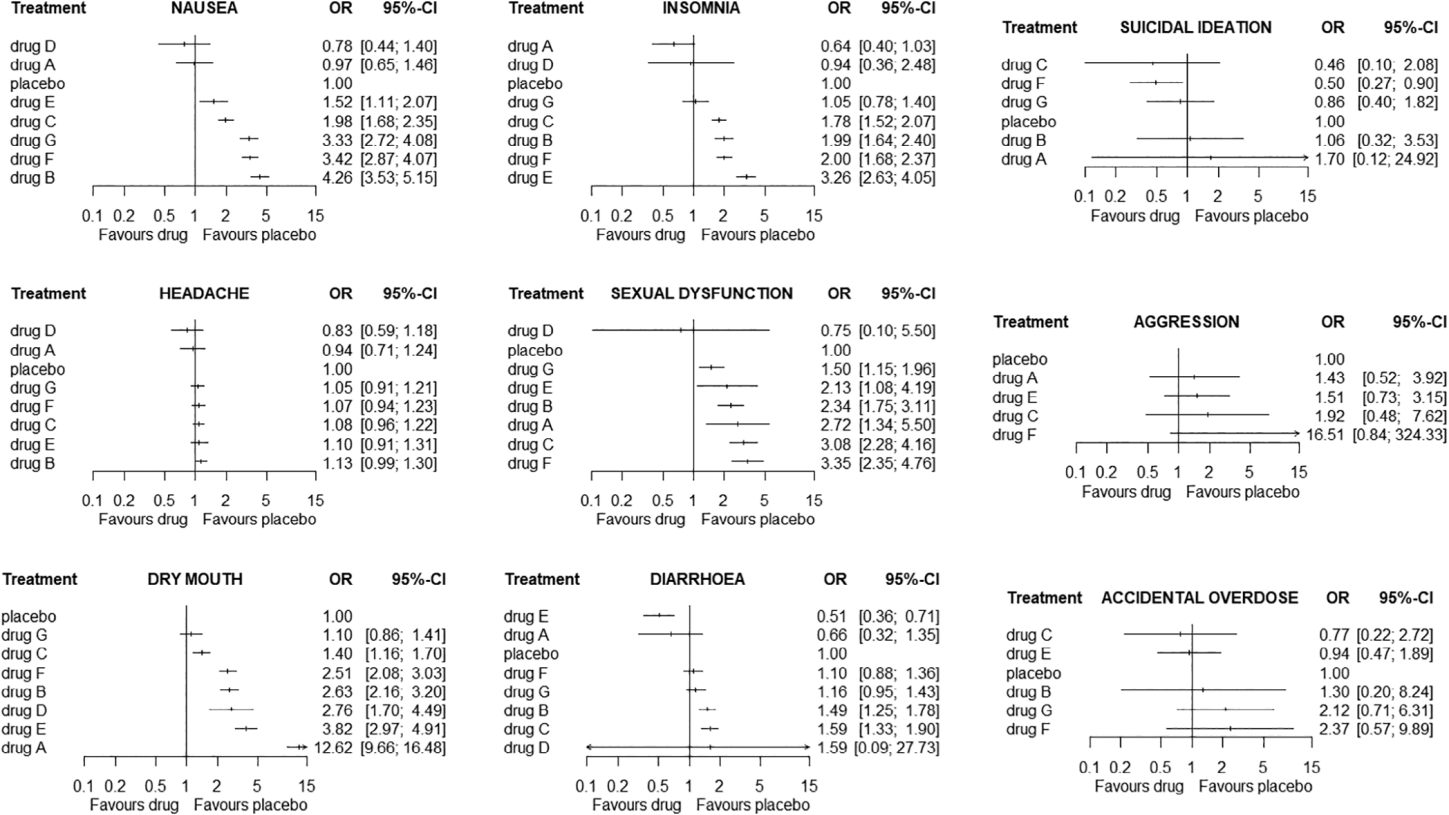
Network meta‐analysis odds ratios for all outcomes, active vs placebo. In each figure, the drugs have been ordered according to the corresponding point estimate of the odds ratios vs placebo

In Figure 1 we present the network graph for one outcome only (ie, nausea). In Figure 2 we show the results of the nine independent NMAs (one per outcome), for each drug vs placebo.

## 3 METHODS

In this section, we describe the procedure we follow in order to draw the Kilim plot. For simplicity, we start by focusing on the case of binary outcomes and then discuss how the graph can be modified to accommodate continuous outcomes as well. The overarching idea is to have a table with a number of rows equal to the number of treatments (rows = treatments), and columns equal to the number of outcomes of interest (columns = outcomes). In the first row, we place the reference treatment in the network, for instance placebo, treatment as usual or standard care. Within each cell of the table (corresponding to a treatment and an outcome), we provide information on the point estimate of the absolute effects of the specific treatment, for the specific outcome. As a visual aid, we color the cells according to the strength of statistical evidence of the relative effect of the treatment vs the reference, for the corresponding outcome. Note that by “strength of statistical evidence” we hereby only refer to the extent to which the estimated effects (taking into account uncertainty) are either in favor or against each treatment, as compared to a baseline treatment. In Section 5, we discuss how the Kilim plot could be modified to instead reflect quality of the evidence. Additional considerations, such as comparisons of the estimated effects to clinically important values can be accommodated in the plot. Below, we describe this procedure in more detail.

### 3.1 Drawing the graph for a binary outcome

*The first step* is to perform NMA for all outcomes. This can be either a frequentist or a Bayesian NMA, using any NMA model, for example, assuming fixed or random effects, using odds ratios or risk ratios, accounting or not for the correlations between the outcomes,^12^ and so on. The output of the first step is estimates of relative treatment effects for all outcomes of interest.

*The second step* is to obtain an estimate of the absolute event rate for each outcome, for the reference intervention. The obvious way to do this is to perform a meta‐analysis of all reference arms in the network. Alternatively, we can use external information (eg, obtained from an observational study) if the information is deemed to be more relevant for clinical practice.

*The third step* is to combine the relative effects from the first step and the reference treatment event rate from the second step in order to estimate the event rates for all treatments in the network. For example, let us assume that we estimated in step one the odds ratio of treatment *X* vs the reference to be $OR_{X}^{\left(S\right)}$ for outcome *S*. Also, assume that for the reference treatment we estimated an event rate equal to $p_{0}^{\left(S\right)}$ in step two. Then, we can estimate the event rate for treatment *X*, outcome *S*, denoted $p_{X}^{\left(S\right)}$ as $p_{X}^{\left(S\right)}=OR_{X}^{\left(S\right)}\mathrm{odd}s_{0}^{\left(S\right)}/\left(1+OR_{X}^{\left(S\right)}\mathrm{odd}s_{0}^{\left(S\right)}\right)$. We follow this method so as to keep intact randomization (ie, relative effects are estimated from regular NMAs) and aiming to obtain an estimate of absolute, rather than relative, effects.

*In the fourth step*, we quantify the strength of statistical evidence, using the *Z*‐score approach. *Z*‐scores are calculated using the estimated effect sizes and standard errors obtained from the NMAs of step one, and possibly taking also into account clinically important values (see Section 3.3). More concretely, let us assume that in Step 1 we performed the NMAs in the odds ratios scale, and we do not wish to further adjust for clinically important values. In that case we simply calculate for treatment *X* and outcome *S*, a *Z*‐score equal to $Z_{X}^{\left(S\right)}=\text{logO}R_{X}^{\left(S\right)}/\mathrm{SE}\left(\text{logO}R_{X}^{\left(S\right)}\right)$. Note that for some of the treatments in the network, we may not have any information regarding outcome *S*. In that case we set the corresponding *Z*‐scores to zero.

*In the fifth step*, we draw the Kilim plot. The cell corresponding to treatment *X*, outcome *S* is colored according to $Z_{X}^{\left(S\right)}$. Let us assume (ie, as in our example of antidepressant dataset) that a negative *Z*‐score is associated with a decrease in side effects when using an active treatment. Then, we associate negative *Z*‐scores with a green color and positive *Z*‐score with a red color. The color becomes darker as $Z_{X}^{\left(S\right)}$ becomes larger (either negative or positive). White areas will correspond to cases where we have limited information ($Z_{X}^{\left(S\right)}$ near zero). This can be because the corresponding confidence intervals are very wide or because a treatment is not included in the network of *S*. Information regarding absolute event rates is shown in the cells, using the results from step three. The plot shows that a treatment is not included in the network for a given outcome by denoting the missing event rate with a dash.

Since *Z*‐scores are related to *P*‐values, and given that the latter are more widely used to communicate uncertainty regarding an estimate, in the legend of the plot we can provide information regarding the coloring scheme in terms of *P*‐values, instead of *Z*‐scores. These are calculated from the *Z*‐scores, using a two‐sided test. Readers should note that the plot will not be affected by the choice between *P*‐values or *Z*‐scores for the legend. Readers should also note that even though we use *P*‐values, we do not recommend dichotomizing results as “statistically significant” or not. We discuss this further in Section 5.

### 3.2 Drawing the graph for a continuous outcome

For a continuous outcome, we can follow the same procedure as above, if the analysis in step one is performed on the natural scale, that is, using mean difference. Then, in step two, we can estimate the mean endpoint result for the reference treatment for each outcome, (or inform it from an external source). In step three, we can estimate the endpoint value for each treatment, using the mean difference vs the reference (from step one) and the mean estimate for the reference treatment (from step two). If the standardized mean difference (SMD) is used (eg, because studies report the outcomes using different scales, and there is no way to convert between the scales), then there is no straightforward way to obtain absolute effects. One possible solution would be to express the SMD as odds ratios using the transformation $\text{lnOR}=\frac{\pi }{\surd 3}\mathrm{SMD}.$ ^13^

### 3.3 Utilizing clinically important values

We can modify the Kilim plot by incorporating clinically important values (CIV) in step four above. Let us assume that step one was conducted using odds ratios. Also assume that we are interested in setting a clinically important value of ${\mathrm{CIV}}_{\mathrm{RD}}^{\left(S\right)}=10\%$ increase on the risk compared to the reference, for outcome *S*. This might be the case when *S* is a relatively minor side effect, and we deem that an increase of up to 10% in the risk of *S* as compared to placebo is acceptable. Then, using $p_{0}^{\left(S\right)}$ and ${\mathrm{CIV}}_{\mathrm{RD}}^{\left(S\right)}$ we can recalculate the corresponding CIV in the odds ratio scale, ${\mathrm{CIV}}_{\text{OR}}^{\left(S\right)}$, and use it to obtain $Z_{X}^{\left(S\right)}=\left(\text{logO}R_{X}^{\left(S\right)}-\mathrm{CI}V_{\text{OR}}^{\left(S\right)}\right)/\mathrm{SE}\left(\text{logO}R_{X}^{\left(S\right)}\right)$. A negative $Z_{X}^{\left(S\right)}$ will imply that *X* does not increase the risk of an event by more than 10% as compared to the reference and positive $Z_{X}^{\left(S\right)}$ will imply that *X* increases the risk by more than 10%. If we instead specify a clinically important value in the log‐odds ratio scale (eg, $\mathrm{CI}V_{\text{OR}}^{\left(S\right)}=\log\left(1.20\right)$), we can directly calculate $Z_{X}^{\left(S\right)}$ using the formula above. Finally, note that the CIVs can be different per outcome.

## 4 KILIM PLOT FOR THE ANTIDEPRESSANTS EXAMPLE

In this section, we present the Kilim plot for the antidepressant data presented in Section 2.

### 4.1 Implementation details

We performed all analyses in R. We ran the NMAs in step one using the netmeta ^14^ package. The analyses were run on the odds ratio scale. We estimated the summary event rate in the placebo arms for each outcome in step two using the metaprop function in meta ^15^ package. The Kilim plot was drawn using the ggplot2 ^16^ package. When coloring the Kilim plot, we truncated the *Z*‐score values. Values that were larger than 3 or smaller than −3 were truncated to 3 and –3, respectively. This was needed to prevent extremely large or small *Z* values dominating one end of the color spectrum. To account for different CIVs per outcome, we developed a web application for our example using the shiny ^17^ package. The web application is available in https://cinema.ispm.unibe.ch/shinies/kilim/. In addition to what we described above, the web application also allows the users to manually set the placebo event rate. This might be useful when different types of patients are deemed to have different baseline risks (eg, when older patients may be more susceptible to a specific type of adverse events).

### 4.2 Results

**FIGURE 3:**
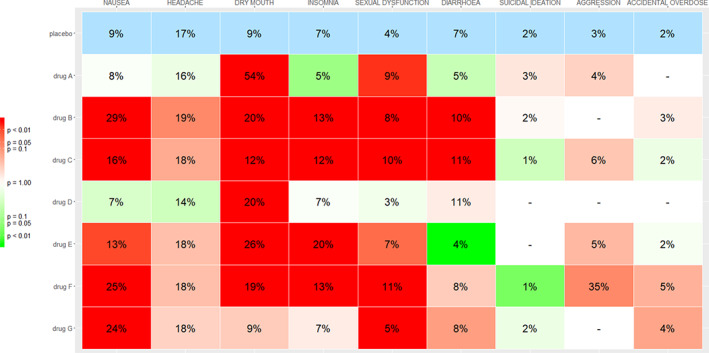
Kilim plot for comparing antidepressant drugs for nine outcomes. The numbers in each cell correspond to the estimated absolute event rates for each outcome and treatment. The colors correspond to the strength of statistical evidence regarding the relative effects vs placebo. A cell with a deep green color indicates strong evidence that the corresponding drug performs better than placebo for the corresponding outcome. Conversely, a deep red cell indicates strong evidence that the drug performs worse than placebo. Colors closer to white indicate lack of evidence on whether the drug performs better or worse than placebo [Colour figure can be viewed at wileyonlinelibrary.com]

In Figure 3, we show the Kilim plot. The plot visualized several aspects of the data and the analysis results. First, the graph provides a quick overview of the treatments being compared for each outcome. For example, for aggression, three of the treatments (drug B, drug D, and drug G) were not compared in the studies; the corresponding cells in the aggression column are denoted with a dash.

Next, the color of the cells facilitates the identification of cases where the statistical evidence for the effect estimate is strong either in favor of the drugs (green colors) or in favor of placebo (red colors). For example, by looking at the column “dry mouth,” we see that there is strong statistical evidence that all drugs perform worse than placebo. Additionally, the numbers in the cells provide an estimate of the absolute effects. This might be particularly important when trying to communicate results to patients. For example, instead of informing a patient that “drug A has an odds ratio of 12.6 versus placebo” (from Figure 2), using the Kilim plot a clinician might instead provide the information that “Without taking the active drug, there is on average 9% probability of having dry mouth. With drug A this probability is 54%.” The corresponding color is dark red, implying that the combination of magnitude and strength of statistical evidence is convincing against drug A. Similarly, most drugs appear to be detrimental for sexual dysfunction, with the estimated event rate ranging from 3% to 11%, whereas in placebo it was 4%. One of the drugs (drug D) has a light green cell, meaning that the corresponding estimate (3% rate) was not strong; thus, this estimate should not play a big role either in favor or against the drug.

Some drugs perform consistently bad for all outcomes, for example, drug B and drug C. On the other hand, drug A and drug D, perform relatively better than the rest of the treatments for most outcomes. In cases when some of the outcomes are of particular importance for a patient, then the decision on how to treat can be guided accordingly, by focusing on the corresponding columns.

**FIGURE 4:**
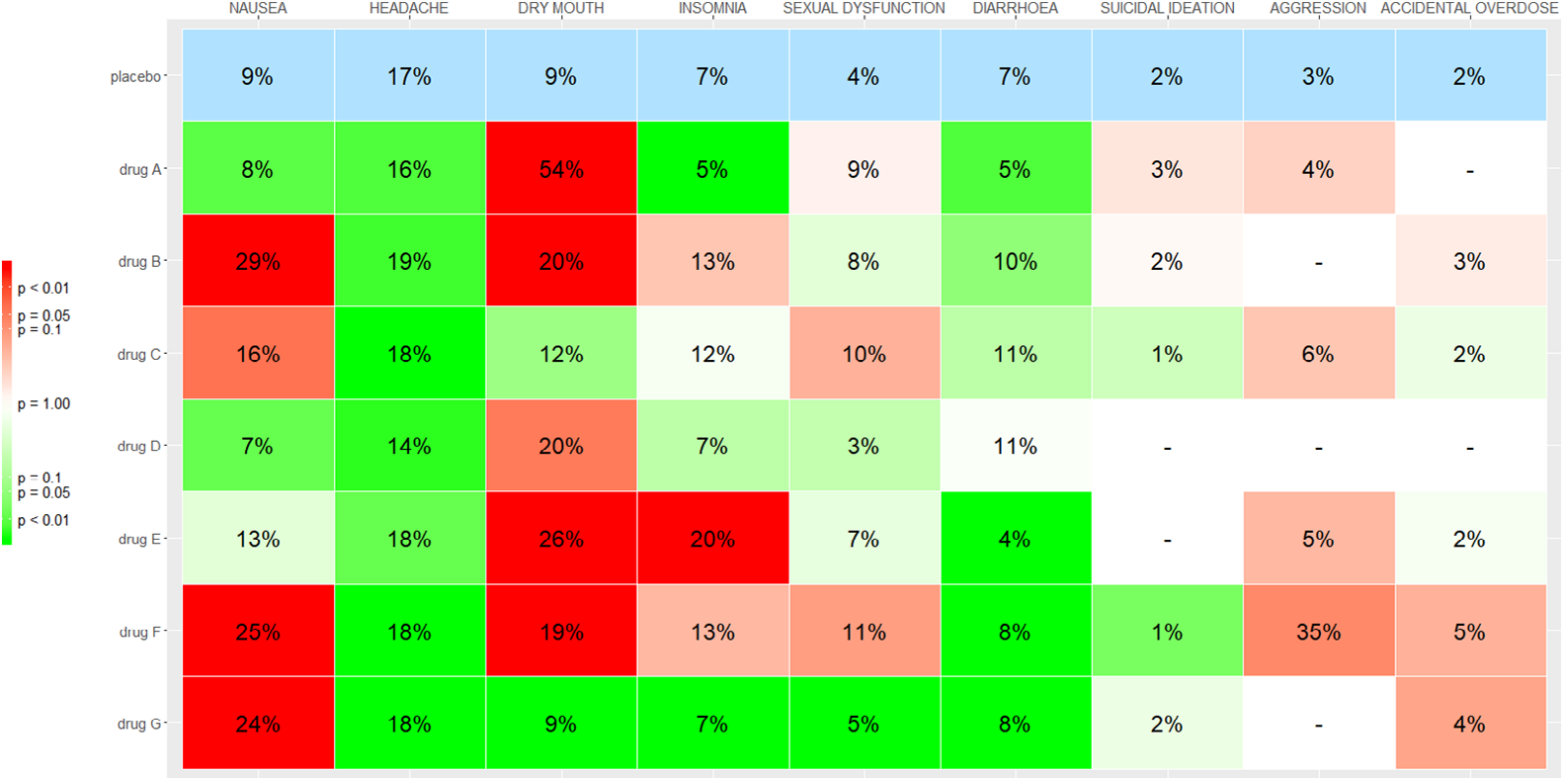
Kilim plot for the antidepressants example, with color adjustment for clinically important values. For nonserious outcomes (ie, all outcomes except suicidal ideation, aggression, accidental overdose) the clinically important value is set to a risk increase of 5%. Dark green cells for these outcomes indicate strong statistical evidence that the corresponding drug leads to an increase of event rate less than 5% as compared to placebo, for the corresponding outcome. Dark red cells indicate strong evidence that the drug increases the event rate by 5% or more. White cells indicate lack of evidence on whether the effects of drugs reach the clinically important values, for example, when the corresponding confidence intervals spans from values smaller than 5% to values larger than 5% [Colour figure can be viewed at wileyonlinelibrary.com]

In Figure 4, we show the Kilim plot from the same dataset, but this time taking into account clinically important values. More specifically, as an illustration we assumed that for the non‐serious adverse events an absolute increase of 5% or less in the event rate may be clinically not so important. For example, an increase of 5% or less as compared to the risk in placebo might not be clinically very meaningful when it comes to nausea. However, for serious adverse events such as suicidal ideation, any increase in the event rate, however small, was deemed to be clinically important. Taking into account this set of clinically important values (ie, nonserious outcomes: risk difference 5%; serious outcomes: risk difference 0%), we draw the Kilim plot of Figure 4. The estimated event rates are the same as in Figure 3; this is because they are based on the same analyses. The only difference regards the colors of the non‐serious outcomes. For instance, by looking at the headache column, we now see that all drugs are colored green. This is because, even though almost all drugs perform worse than placebo, no drug is estimated to lead to an increase of 5% or more in the event rates. The maximum increase in risk is seen for drug B, and it is equal to 2%; according to our rule, this difference is deemed to be clinically unimportant. Using this graph, a clinician or a patient might also decide to consider drug G, in addition to drug A and drug D. The full decision will of course need to be made by looking at efficacy outcomes, not included in the data we used for illustration.

Figure 4 can be redrawn using different thresholds for the clinically important value of each different outcome. For example, a patient might accept being prescribed a drug with an average 10% increase in the probability of dry mouth or diarrhea, but might have zero tolerance (ie, clinically important value of 0%) for insomnia and nausea. In addition, this patient might belong to a high‐risk group regarding aggression, so that the corresponding baseline probability of an event is deemed to be 10% instead of 3%. Our web app can easily facilitate such information, to draw a personalized Kilim plot.

## 5 DISCUSSION

In this article, we have introduced a new graphical tool for visualizing results from multiple outcomes NMA, the Kilim plot. The aim of the Kilim plot is to provide a holistic view of the available evidence expressed in terms of absolute treatment effects and their corresponding strength of statistical evidence.

First, the plot provides information regarding the estimated absolute effects for the baseline treatment in the network, for all outcomes. These are estimated from analyses of the baseline arms in all studies. Second, the plot provides information on the absolute effects for all non‐baseline treatments. These are estimated using the results from the NMA (ie, relative effects) and the estimated effects for the baseline treatment, for each outcome. Furthermore, using a coloring scheme, the Kilim plot provides information on which treatments there is strong statistical evidence that they have a beneficial effect (deep green colors) and on which treatments there is strong evidence that they have a detrimental effect (deep red colors), for each outcome. Additionally, the Kilim plot incorporates features which are potentially useful to clinicians and patients, that is, it can include considerations regarding clinically important values and it allows users to input baseline event rates.

The Kilim plot does not provide information on relative effects, for example, odds ratios or risk differences. Also, it does not show confidence intervals; instead it uses a coloring scheme to communicate uncertainty. Thus, the Kilim plot can be used to augment (but not replace) the usual methods of presenting results from NMA, such as league tables and plots of relative effects vs a reference treatment (as in Figure 2). Our plot shares some similarities with the rank‐heat plot,^4^ that is, both are methods for visualizing results from multiple outcomes NMA. However, the Kilim plot aims at providing information on absolute effects, while the rank‐heat plot is focused on ranking metrics (eg, SUCRAs). Moreover, the Kilim plot can visualize the evidence with respect to clinically important values.

The reason why we included information on absolute rather than relative effect in the graph is because the latter are usually hard to interpret and utilize in every‐day clinical practice, especially for binary outcomes. For example, a risk ratio of 2.5 can have very different implications, depending on whether the baseline event rate is 1% or 10%; moreover, most people would have a hard time understanding the meaning of an odds ratio of 1.7, let alone use multiple odds ratios regarding different outcomes to make a clinical decision among competing treatments. Readers should note at this point that the Kilim plot bears similarities with the summary of findings table in Cochrane reviews.^13^ Such a table summarizes results of a systematic review by showing measures of absolute effects without the intervention, measures of absolute effects with the intervention, and also relative treatment effects.

Our method keeps intact randomization (ie, relative effects are estimated from regular NMAs) and aims to obtain an estimate of absolute, rather than relative, effects. Alternatively, a so‐called “arm‐based” NMA model^18^ could have been used in step one of Section 3.1, to directly estimate event rates. Arm‐based models, however, have received some criticism^19^; thus, we here presented how to estimate absolute event rates using the usual “contrast‐based” models.

Our readers should also note that although we use P‐values to communicate uncertainty, we do not use or promote the concept of “statistical significance,” that is, we have avoided dichotomizing the evidence as significant or not according to an arbitrary threshold, such as *P*‐value = .05 or any other value. Statistical significance has been a target for much criticism in science in general,^20^ and especially for NMA.^21^ We hereby also recommend meta‐analysts to avoid using it to characterize NMA results.

One limitation of our approach is that in order to estimate absolute event rates, we assume that relative effects are independent of the absolute effects for the reference treatment. For example, we described how we first estimate odds ratios using a regular NMA, and then how we apply these odds ratios to a baseline risk (ie, risk in placebo), to estimate absolute event rates for all treatments. Information on the baseline risk can be obtained using an external source of information. However, odds ratios may be correlated to baseline risk, for example, when a drug works better in more severely ill patients. In such cases, our approach will lead to biased estimates; in these scenarios it might be advisable to avoid using external sources of information for estimating the baseline risk.

To further facilitate decision‐making, the Kilim plot can be modified to account for clinically important values. For example, we might have strong evidence that a drug increases the probability of headache by 1% (confidence interval 0.5%‐2.5%) as compared to placebo. However, this small increase might be clinically irrelevant, despite its strong statistical evidence. In this article, we have discussed how such considerations can be incorporated in such a plot, by changing the coloring scheme. Other possible extensions for the Kilim plot would be to show confidence intervals for the event rates; to use different font sizes for the event rates, depending on the precision of the relative effects; to group treatments by class; to indicate in the plot whether the estimates for each treatment vs the baseline were derived from direct, indirect, or mixed evidence; to group outcomes by their type, for example, efficacy/acceptability/safety; to use different coloring schemes for different classes of outcomes; to color the cells after using the CINEMA approach^22^ to assess the quality of the evidence (online tool available in https://cinema.ispm.unibe.ch/). We hereby did not pursue such extensions, aiming to keep the plot as simple as possible.

To illustrate the Kilim plot we utilized a dataset from depression, where seven drugs and placebo were compared for nine different outcomes. Although our example only used dichotomous outcomes, an extension of the plot for continuous outcomes would be straightforward. All codes needed to produce the Kilim plot are provided in the Appendix and online. In addition, we developed an example web application using R shiny, to illustrate how the graph can be adjusted for different choices of clinically important values and/or different baseline risks. The code for the web app can also be found in the Appendix and online.

In summary, we believe that the Kilim plot can be a valuable aid in summarizing and communicating results from NMAs on multiple outcomes. It can be especially useful for larger networks, for the case of many outcomes, and when aiming to communicate NMA results with patients and/or clinicians, so as to facilitate every‐day clinical practice.

## CONFLICT OF INTEREST

T.A.F. reports personal fees from Mitsubishi‐Tanabe, MSD, and Shionogi, and a grant from Mitsubishi‐Tanabe, outside the submitted work; T.A.F. has a patent 2018‐177688 pending. T.P. has participated in speaker meetings organised by Sunovion and Lundbeck. G.S. was invited and participated in two scientific meetings about the role of real‐world evidence (not related to the work presented in this article), organized by Biogen and Merck. A.C. reports research and consultancy fees from INCiPiT (Italian Network for Paediatric Trials), CARIPLO Foundation, and Angelini Pharma, outside the submitted work; A.C. has also organized a workshop about digital mental health sponsored by Angelini Pharma. The other authors report no conflicts of interest.

## Supporting information


**Appendix S1:** Supplementary Information

## Data Availability

Data sharing is not applicable to this article as no new data were created in this study.
