# Supplementary material for: The Kilim plot: A tool for visualizing network meta‐analysis results for multiple outcomes
Source: Res Synth Methods. 2020 Jul 16;12(1):86–95. doi: 10.1002/jrsm.1428 (PMC7818463; doi:10.1002/jrsm.1428)
Supplement: Supplementary file 1 — Appendix S1: Supplementary Information [file JRSM-12-86-s001.docx]

**Appendix for “The Kilim plot: a tool for visualizing network meta-analysis results for multiple outcomes”**

Michael Seo^1^, Toshi A. Furukawa^2^, Areti Angeliki Veroniki^3,4,5^, Toby Pillinger^6,7^, Anneka Tomlinson^8^, Georgia Salanti^1^, Andrea Cipriani^8^, Orestis Efthimiou^1^

*^1^Institute of Social and Preventive Medicine, University of Bern, Bern, Switzerland*

*^2^Departments of Health Promotion and Human Behavior and of Clinical Epidemiology, Kyoto University Graduate School of Medicine / School of Public Health, Kyoto, Japan*

*^3^Department of Primary Education, School of Education, University of Ioannina, loannina, Greece*

*^4^Knowledge Translation Program, Li Ka Shing Knowledge Institute, St. Michael’s Hospital, Unity Health Toronto, Toronto, ON, Canada*

*^5^Institute of Reproductive and Developmental Biology, Department of Surgery & Cancer, Faculty of Medicine, Imperial College, London, United Kingdom*

*^6^Institute of Psychiatry, Psychology and Neuroscience, King’s College London, London, UK*

*^7^MRC London Institute of Medical Sciences, Faculty of Medicine, Imperial College London, Hammersmith Hospital Campus, London, UK*

*^8^Department of Psychiatry, University of Oxford, Oxford, United Kingdom*

# Example code for drawing Kilim plot

## Generating example dataset

Here we generate a mock dataset with 5 outcomes and 7 treatments

library(ggplot2)

library(netmeta)

# make a function that generates data using logOR and range for probability of an event in treatment 1 (i.e. p.ref.1 = lower bound and p.ref.2 = upper bound)

generateData <- function(logOR = NULL, p.ref.1, p.ref.2){

ntreat <- 7

t1 <- rep(combn(7,2)[1,], each = 2) # arm 1 treatment

t2 <- rep(combn(7,2)[2,], each = 2) # arm 2 treatment

nstudy <- length(t1) # number of studies

OR <- exp(logOR)

studlab <- seq(nstudy)

n1 <- n2 <- round(runif(nstudy,50,100)) # sample size for each study

# study-specific probability of an event in treatment 1

p.ref <- runif(nstudy,p.ref.1, p.ref.2)

odds.ref <- p.ref / (1 - p.ref)

# define probabilities per treatment, per study arm

odds.t1 <- odds.t2 <- r1 <- r2 <- vector()

for(j in 1:nstudy){

odds.t1[j] <- odds.ref[j] * OR[t1[j]]

odds.t2[j] <- odds.ref[j] * OR[t2[j]]

}

p.t1 <- odds.t1/(1+odds.t1)

p.t2 <- odds.t2/(1+odds.t2)

for(j in 1:nstudy){

r1[j] <- rbinom(1, n1[j], p.t1[j])

r2[j] <- rbinom(1, n2[j], p.t2[j])

}

data01 <- data.frame(studlab = studlab, drug = t1, outcome = r1, n = n1)

data02 <- data.frame(studlab = studlab, drug = t2, outcome = r2, n = n2)

data <- rbind(data01, data02)

data <- data[order(data$studlab, data$drug),]

rownames(data) <- 1:dim(data)[1]

return(data)

}

# generate different data for 5 outcomes

data1 <- generateData(logOR = c(0, 0.1, 0.2, 0.3, 0.4, 0.5, 0.6), 0.1, 0.15)

data2 <- generateData(logOR = c(0, -0.1, -0.2, -0.3, -0.8, -0.9, -1.2), 0.15, 0.20)

data3 <- generateData(logOR = c(0, -3, 2, -0.5, 0.3, -0.5, 0), 0.10, 0.12)

data4 <- generateData(logOR = c(0, 0.2, 0.3, 0, 0, 0.2, 0.1), 0.05, 0.07)

data5 <- generateData(logOR = c(0, -0.2, -0.3, -0.4, -0.1, 0, 0), 0.10, 0.20)

store0 <- list(data1, data2, data3, data4, data5)

## Performing the analysis

# Fit network meta analysis

p1 = pairwise(treat = drug, event = outcome, n = n, studlab = studlab, data = data1, sm = "OR", allstudies=T)

result1 <- netmeta(p1)

p2 = pairwise(treat = drug, event = outcome, n = n, studlab = studlab, data = data2, sm = "OR", allstudies=T)

result2 <- netmeta(p2)

p3 = pairwise(treat = drug, event = outcome, n = n, studlab = studlab, data = data3, sm = "OR", allstudies=T)

result3 <- netmeta(p3)

p4 = pairwise(treat = drug, event = outcome, n = n, studlab = studlab, data = data4, sm = "OR", allstudies=T)

result4 <- netmeta(p4)

p5 = pairwise(treat = drug, event = outcome, n = n, studlab = studlab, data = data5, sm = "OR", allstudies=T)

result5 <- netmeta(p5)

store <- list(result1, result2, result3, result4, result5)

# Make a data frame with values needed for plotting

final <- data.frame()

for(i in 1:5){

data <- store0[[i]]

net1 <- store[[i]]

# treatment estimate (odds ratio) from netmeta

OR.pla <- data.frame("drug" = colnames(net1$TE.random))

OR.pla$logOR <- net1$TE.random[,1]

OR.pla$seTE <- net1$seTE.random[,1]

OR.pla$OR <- exp(OR.pla$logOR)

OR.pla <- OR.pla[-which(OR.pla$drug == 1),] # exclude comparison with placebo itself which is 0

# meta analysis of event rates in placebo

meta.pla = metaprop(event = round(data$outcome[data$drug==1]), n = data$n[data$drug==1], method = "GLMM")

rate.pla = exp(meta.pla$TE.fixed)/(1+exp(meta.pla$TE.fixed))

odds.pla=rate.pla/(1-rate.pla)

# calculate event rate for treatment

OR.pla$event.rate <- round(OR.pla$OR*odds.pla/(1+OR.pla$OR*odds.pla),digits=3)

# Calculate Zscore accounting for clinically important risk difference

clinically.important.RD.0 <- 0.0

risk.drugs.0 <- clinically.important.RD.0+rate.pla

OR.import.0 <- risk.drugs.0/(1-risk.drugs.0)/((rate.pla)/(1-rate.pla))

OR.pla$Zscore.0 <- (OR.pla$logOR-log(OR.import.0))/OR.pla$seTE

outcome.result <- data.frame(outcome = paste("outcome", i), drug = OR.pla$drug, Zscore = OR.pla$Zscore.0, event.rate = round(OR.pla$event.rate*100,1), rate.pla = rate.pla, logOR = OR.pla$logOR, seTE = OR.pla$seTE)

final <- rbind(final, outcome.result)

}

final_data <- final

final_data <- final_data[,c("outcome", "drug", "Zscore", "event.rate")]

#add in control treatment (i.e. treatment = 1)

add_placebo <- data.frame(outcome = paste("outcome", 1:5), drug = rep(1, 5), Zscore = rep(NA, 5), event.rate = round(unique(final$rate.pla)*100,1) )

final_data <- rbind(final_data, add_placebo)

final_data$Zscore2 <- final_data$Zscore #truncated zscore

final_data$Zscore2[final_data$Zscore2 < -3] = -3

final_data$Zscore2[final_data$Zscore2 > 3] = 3

add_percent <- function(x){if(!is.na(x)){paste0(x, "%")} else{x}}

final_data$event.rate <- sapply(final_data$event.rate, add_percent)

# order the drugs accordingly

final_data$drug <- paste("treatment", final_data$drug)

final_data$drug <- factor(final_data$drug, level = paste("treatment", 7:1), ordered = TRUE)

## Drawing Kilim plot

ggplot(final_data, aes(outcome, drug)) +

geom_tile(aes(fill = round(Zscore2,2)), colour = "white") +

geom_text(aes(label= event.rate), size = 6) +

scale_fill_gradient2(low = "green", mid = "white", high = "red", na.value = "lightskyblue1", breaks = c(-2.575829, -1.959964, -1.644854, 0, 1.644854, 1.959964, 2.575829), limits = c(-3, 3), labels = c("p < 0.01", "p = 0.05", "p = 0.1", "p = 1.00", "p = 0.1", "p = 0.05","p < 0.01"))+

guides(fill = guide_colourbar(barwidth = 0.5, barheight = 15)) +

labs(x = "",y = "") +

theme(legend.title = element_blank(),axis.text.x = element_text(size = 12),axis.text.y = element_text(size = 12),legend.position = "left", legend.text = element_text(size = 12)) +

scale_x_discrete(position = "top")

# Code for shiny app

In the shiny app, Z-score is calculated each time the user updates the clinically important values.

library("shiny")

library("ggplot2")

library("netmeta")

generateData <- function(logOR = NULL, p.ref.1, p.ref.2){

ntreat <- 7

t1 <- rep(combn(7,2)[1,], each = 2) # arm 1 treatment

t2 <- rep(combn(7,2)[2,], each = 2) # arm 2 treatment

nstudy <- length(t1) # number of studies

OR <- exp(logOR)

studlab <- seq(nstudy)

n1 <- n2 <- round(runif(nstudy,50,100)) # sample size for each study

# study-specific probability of an event in treatment 1

p.ref <- runif(nstudy,p.ref.1, p.ref.2)

odds.ref <- p.ref / (1 - p.ref)

# define probabilities per treatment, per study arm

odds.t1 <- odds.t2 <- r1 <- r2 <- vector()

for(j in 1:nstudy){

odds.t1[j] <- odds.ref[j] * OR[t1[j]]

odds.t2[j] <- odds.ref[j] * OR[t2[j]]

}

p.t1 <- odds.t1/(1+odds.t1)

p.t2 <- odds.t2/(1+odds.t2)

for(j in 1:nstudy){

r1[j] <- rbinom(1, n1[j], p.t1[j])

r2[j] <- rbinom(1, n2[j], p.t2[j])

}

data01 <- data.frame(studlab = studlab, drug = t1, outcome = r1, n = n1)

data02 <- data.frame(studlab = studlab, drug = t2, outcome = r2, n = n2)

data <- rbind(data01, data02)

data <- data[order(data$studlab, data$drug),]

rownames(data) <- 1:dim(data)[1]

return(data)

}

# generate different data for 5 outcomes

data1 <- generateData(logOR = c(0, 0.1, 0.2, 0.3, 0.4, 0.5, 0.6), 0.1, 0.15)

data2 <- generateData(logOR = c(0, -0.1, -0.2, -0.3, -0.8, -0.9, -1.2), 0.15, 0.20)

data3 <- generateData(logOR = c(0, -3, 2, -0.5, 0.3, -0.5, 0), 0.10, 0.12)

data4 <- generateData(logOR = c(0, 0.2, 0.3, 0, 0, 0.2, 0.1), 0.05, 0.07)

data5 <- generateData(logOR = c(0, -0.2, -0.3, -0.4, -0.1, 0, 0), 0.10, 0.20)

store0 <- list(data1, data2, data3, data4, data5)

# Fit network meta analysis

p1 = pairwise(treat = drug, event = outcome, n = n, studlab = studlab, data = data1, sm = "OR", allstudies=T)

result1 <- netmeta(p1)

p2 = pairwise(treat = drug, event = outcome, n = n, studlab = studlab, data = data2, sm = "OR", allstudies=T)

result2 <- netmeta(p2)

p3 = pairwise(treat = drug, event = outcome, n = n, studlab = studlab, data = data3, sm = "OR", allstudies=T)

result3 <- netmeta(p3)

p4 = pairwise(treat = drug, event = outcome, n = n, studlab = studlab, data = data4, sm = "OR", allstudies=T)

result4 <- netmeta(p4)

p5 = pairwise(treat = drug, event = outcome, n = n, studlab = studlab, data = data5, sm = "OR", allstudies=T)

result5 <- netmeta(p5)

store <- list(result1, result2, result3, result4, result5)

# Make a data frame with values needed for plotting

final <- data.frame()

for(i in 1:5){

data <- store0[[i]]

net1 <- store[[i]]

# treatment estimate (odds ratio) from netmeta

OR.pla <- data.frame("drug" = colnames(net1$TE.random))

OR.pla$logOR <- net1$TE.random[,1]

OR.pla$seTE <- net1$seTE.random[,1]

OR.pla$OR <- exp(OR.pla$logOR)

OR.pla <- OR.pla[-which(OR.pla$drug == 1),] #exclude comparison with placebo itself which is 0

# meta analysis of event rates in placebo

meta.pla = metaprop(event = round(data$outcome[data$drug==1]), n = data$n[data$drug==1], method = "GLMM")

rate.pla = exp(meta.pla$TE.fixed)/(1+exp(meta.pla$TE.fixed))

odds.pla=rate.pla/(1-rate.pla)

# calculate event rate for treatment

OR.pla$event.rate <- round(OR.pla$OR*odds.pla/(1+OR.pla$OR*odds.pla),digits=3)

# Calculate Zscore accounting for clinically important risk difference

clinically.important.RD.0 <- 0.0

risk.drugs.0 <- clinically.important.RD.0+rate.pla

OR.import.0 <- risk.drugs.0/(1-risk.drugs.0)/((rate.pla)/(1-rate.pla))

OR.pla$Zscore.0 <- (OR.pla$logOR-log(OR.import.0))/OR.pla$seTE

outcome.result <- data.frame(outcome = paste("outcome", i), drug = OR.pla$drug, OR = OR.pla$OR ,Zscore = OR.pla$Zscore.0, event.rate = round(OR.pla$event.rate*100), rate.pla = rate.pla, logOR = OR.pla$logOR, seTE = OR.pla$seTE)

final <- rbind(final, outcome.result)

}

# Now the shiny part begins

ui <- shinyUI(fluidPage(

shinyjs::inlineCSS(list(body = "color:DarkBlue")),

titlePanel(h1("The Kilim plot: a tool for visualizing network meta-analysis results for multiple outcomes")),

tabPanel("Heatplot", plotOutput("plot1")),

hr(),

h3("Clinically important value (in risk difference)"),

fluidRow(

column(3,

sliderInput("outcome1", "outcome1:", min = 0, max = 0.1, value = 0, step = 0.01),

sliderInput("outcome2", "outcome2:", min = 0, max = 0.1, value = 0, step = 0.01)

),

column(4, offset = 1,

sliderInput("outcome3", "outcome3:", min = 0, max = 0.1, value = 0, step = 0.01),

sliderInput("outcome4", "outcome4:", min = 0, max = 0.1, value = 0, step = 0.01)

),

column(4,

sliderInput("outcome5", "outcome5:", min = 0, max = 0.1, value = 0, step = 0.01)

)

),

hr(),

h3("Absolute event rate for the reference intervention (external information; if none, set it to 0)"),

fluidRow(

column(3,

sliderInput("outcome1_e", "outcome1:", min = 0, max = 0.99, value = 0, step = 0.01),

sliderInput("outcome2_e", "outcome2:", min = 0, max = 0.99, value = 0, step = 0.01)

),

column(4, offset = 1,

sliderInput("outcome3_e", "outcome3:", min = 0, max = 0.99, value = 0, step = 0.01),

sliderInput("outcome4_e", "outcome4:", min = 0, max = 0.99, value = 0, step = 0.01)

),

column(4,

sliderInput("outcome5_e", "outcome5:", min = 0, max = 0.99, value = 0, step = 0.01)

)

)

))

server <- shinyServer(function(input, output) {

rate.pla.list <- unique(final$rate.pla)

getData <- reactive({

if(input$outcome1_e != 0){

rate.pla.list[1] <- input$outcome1_e

}

if(input$outcome2_e != 0){

rate.pla.list[2] <- input$outcome2_e

}

if(input$outcome3_e != 0){

rate.pla.list[3] <- input$outcome3_e

}

if(input$outcome4_e != 0){

rate.pla.list[4] <- input$outcome4_e

}

if(input$outcome5_e != 0){

rate.pla.list[5] <- input$outcome5_e

}

rate.pla <- rep(rate.pla.list, table(final$outcome))

odds.pla=rate.pla/(1-rate.pla)

# calculate event rate for treatment using the formula

final$event.rate <- round(final$OR* odds.pla/ (1+final$OR*odds.pla) * 100)

# constantly update clinically important values

clinically.important.RD.list <- c(input$outcome1, input$outcome2, input$outcome3, input$outcome4, input$outcome5)

# calculate adjusted z-score

Zscore <- vector()

for(i in 1:length(rate.pla.list)){

rate.pla <- rate.pla.list[i]

clinically.important.RD <- clinically.important.RD.list[i]

risk.drugs <- clinically.important.RD + rate.pla

OR.import <- risk.drugs/(1-risk.drugs)/((rate.pla)/(1-rate.pla))

logOR <- final[final$outcome == paste("outcome", i), "logOR"]

seTE <- final[final$outcome == paste("outcome", i), "seTE"]

Zscore <- c(Zscore, (logOR-log(OR.import))/seTE)

}

final_data <- final

final_data$Zscore <- Zscore

final_data <- final_data[,c("outcome", "drug", "Zscore", "event.rate")]

#add in placebo arms

placebo.arms <- data.frame(outcome = paste("outcome", 1:5), drug = rep(1, 5), Zscore = rep(NA, 5), event.rate = round(rate.pla.list*100) )

final_data <- rbind(final_data, placebo.arms)

final_data$Zscore2 <- final_data$Zscore #truncated zscore

final_data$Zscore2[final_data$Zscore2 < -3] = -3

final_data$Zscore2[final_data$Zscore2 > 3] = 3

add_percent <- function(x){if(!is.na(x)){paste0(x, "%")} else{x}}

final_data$event.rate <- sapply(final_data$event.rate, add_percent)

# order the drugs accordingly

final_data$drug <- paste("treatment", final_data$drug)

final_data$drug <- factor(final_data$drug, level = paste("treatment", 7:1), ordered = TRUE)

# fill in missing combination

dat2 <- with(final_data, expand.grid(outcome = levels(outcome), drug = levels(drug)))

final_data2 <- merge(final_data, dat2, all.y = TRUE)

final_data2[final_data2$drug != "treatment 1" & is.na(final_data2$Zscore), "Zscore2"] <- 0

final_data2[final_data2$drug != "treatment 1" & is.na(final_data2$Zscore), "event.rate"] <- "-"

return(final_data2)

})

output$plot1 = renderPlot({

final_data = getData()

ggplot(final_data, aes(outcome, drug)) +

geom_tile(aes(fill = round(Zscore2,2)), colour = "white") +

geom_text(aes(label= event.rate), size = 6) +

scale_fill_gradient2(low = "green", mid = "white", high = "red", na.value = "lightskyblue1", breaks = c(-2.575829, -1.959964, -1.644854, 0, 1.644854, 1.959964, 2.575829), limits = c(-3, 3), labels = c("p < 0.01", "p = 0.05", "p = 0.1", "p = 1.00", "p = 0.1", "p = 0.05","p < 0.01"))+

guides(fill = guide_colourbar(barwidth = 0.5, barheight = 15)) +

labs(x = "",y = "") +

theme(legend.title = element_blank(),axis.text.x = element_text(size = 12),axis.text.y = element_text(size = 12),legend.position = "left", legend.text = element_text(size = 12)) +

scale_x_discrete(position = "top")

})

})

shinyApp(ui = ui, server = server)
